# Functional and Metagenomic Evaluation of Ibezapolstat for Early Evaluation of Anti-Recurrence Effects in Clostridioides difficile Infection

**DOI:** 10.1128/aac.02244-21

**Published:** 2022-07-06

**Authors:** Jacob McPherson, Chenlin Hu, Khurshida Begum, Weiqun Wang, Chris Lancaster, Anne J. Gonzales-Luna, Caroline Loveall, Michael H. Silverman, M. Jahangir Alam, Kevin W. Garey

**Affiliations:** a University of Houston College of Pharmacy, Houston, Texas, USA; b Acurx Pharmaceuticals, Staten Island, New York, USA

**Keywords:** *Clostridioides difficile*, clinical trial, human subjects, metagenomics, bile acids

## Abstract

Reduction of Clostridioides difficile infection (CDI) recurrence is an essential endpoint for CDI-directed antibiotic development that is often not evaluated until Phase III trials. The purpose of this project was to use a functional and metagenomic approach to predict the potential anti-CDI recurrence effect of ibezapolstat, a DNA polymerase IIIC inhibitor, in clinical development for CDI. As part of the Phase I ibezapolstat clinical study, stool samples were collected from 22 healthy volunteers, who were given either ibezapolstat or vancomycin. Stool samples were evaluated for microbiome changes and bile acid concentrations. Ibezapolstat 450 mg and vancomycin, but not ibezapolstat 300 mg, showed statistically significant changes in alpha diversity over time compared to that of a placebo. Beta diversity changes confirmed that microbiota were significantly different between study groups. Vancomycin had a more wide-ranging effect on the microbiome, characterized by an increased proportion of Gammaproteobacteria. Ibezapolstat demonstrated an increased proportion of Actinobacteria, including the Bifidobacteriaceae family. Using a linear regression analysis, vancomycin was associated with significant increases in primary bile acids as well as primary:secondary bile acid ratios. An overabundance of Enterobacteriaceae was most highly correlated with primary bile acid concentrations (*r* = 0.63; *P* < 0.0001). Using Phase I healthy volunteer samples, beneficial changes suggestive of a lower risk of CDI recurrence were associated with ibezapolstat compared to vancomycin. This novel omics approach may allow for better and earlier prediction of anti-CDI recurrence effects for antibiotics in the clinical development pipeline.

## INTRODUCTION

Clostridioides difficile infection (CDI) is the most common cause of infectious gastroenteritis in hospitalized patients and the most common cause of death due to gastroenteritis in the United States of America (1). The pathophysiology of CDI includes disruption of the healthy gut microbiome, usually with high-risk antibiotics (2). Oral vancomycin, the antibiotic most commonly used to treat CDI, is effective at killing vegetative C. difficile but disrupts the microbiota, leading to a high rate of recurrence after the end of antibiotic therapy (3). A key change in the microbiome that increases the risk of CDI and recurrent CDI is decreasing the abundance and diversity of microbiota, including key bacterial species responsible for conversion of primary bile acids to secondary bile acids, in the gut. This dysbiosis allows the germination of C. difficile spores, which are ubiquitous in the environment, to cause disease (4). Ideally, a new drug in development would display similarly potent activity against C. difficile but would not have activity against key host microbiota preventing dysbiosis and would not allow for further germination and infection by C. difficile once therapy is completed (5).

Two recent Phase III clinical trials highlight the importance of understanding the pathophysiology of C. difficile recurrence and antibiotic pharmacology earlier in the drug development process (6–11). Cadazolid, a novel, nonabsorbable antibiotic primarily targeting Gram-positive Firmicute or Actinobacteria phyla, has a minimal effect on Bacteroidetes (6), and thus has a narrower spectrum than vancomycin. Positive Phase II clinical trials led to a large Phase III trial, in which a sustained clinical cure was not observed (7). Surotomycin, a cyclic lipopeptide, had a similar spectrum of activity as cadazolid and similar positive Phase II clinical trial results (8, 9). However, a sustained clinical response difference was not observed in the Phase III clinical trial (10, 11). Although each of these two antibiotics had a minimal effect on host microbiota, in particular, the phylum Bacteroidetes, more advanced microbiome evaluations were not performed during the clinical trial drug development process.

Ibezapolstat is a Gram-positive selective spectrum (GPSS) DNA polymerase IIIC inhibitor currently in the clinical trial drug development process, having completed Phase I healthy volunteer studies (12). The design for the Phase I study included a comparator arm with vancomycin and daily stool samples collected for microbiome analysis. This provided a unique opportunity to develop an approach to assess the possible anti-recurrence effect of ibezapolstat using the known pathophysiology of C. difficile recurrence. The goals of this study were to assess the microbiome (taxa, alpha, and beta diversity) changes as well as the bile acid changes associated with ibezapolstat compared to those associated with vancomycin by using samples obtained from the Phase I healthy volunteer study.

## RESULTS

### Description of clinical trial.

Twenty-two subjects (female: 33%) aged 30 ± 8 years were enrolled. Six patients each were given either vancomycin, ibezapolstat 300 mg, or ibezapolstat 450 mg, and an additional four were given a placebo. A full description of the Phase I study, including safety, food effects, pharmacokinetics, and initial metagenomic analyses, has been described previously (12).

### Metagenomic analysis.

**FIG 1:**
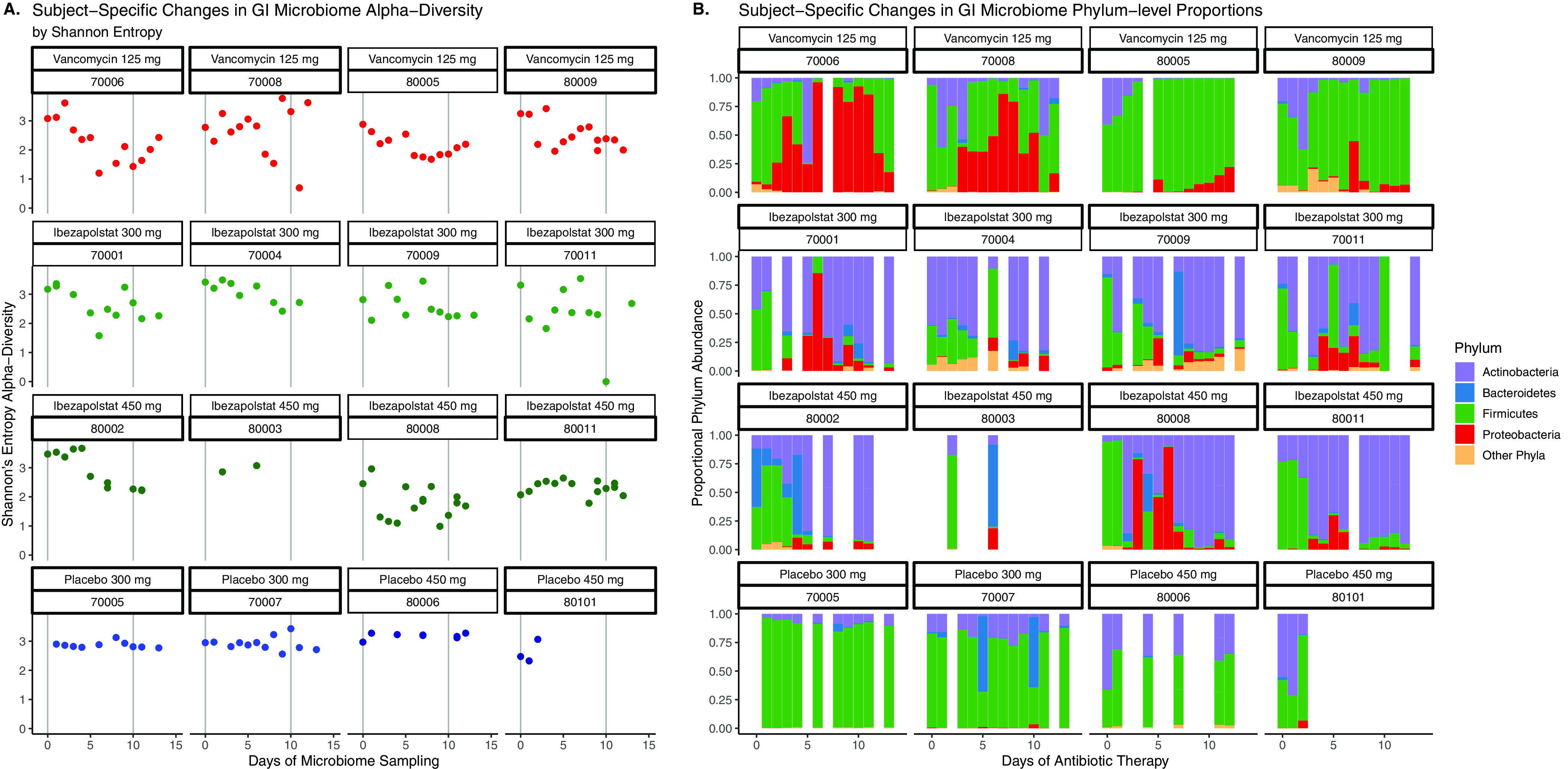
Subject specific changes in alpha diversity (A) and phylum-level proportions (B) during the study time period. Each box represents 1 patient given a 10-day course of the study drug or a placebo over a 13-day study period.

**FIG 2:**
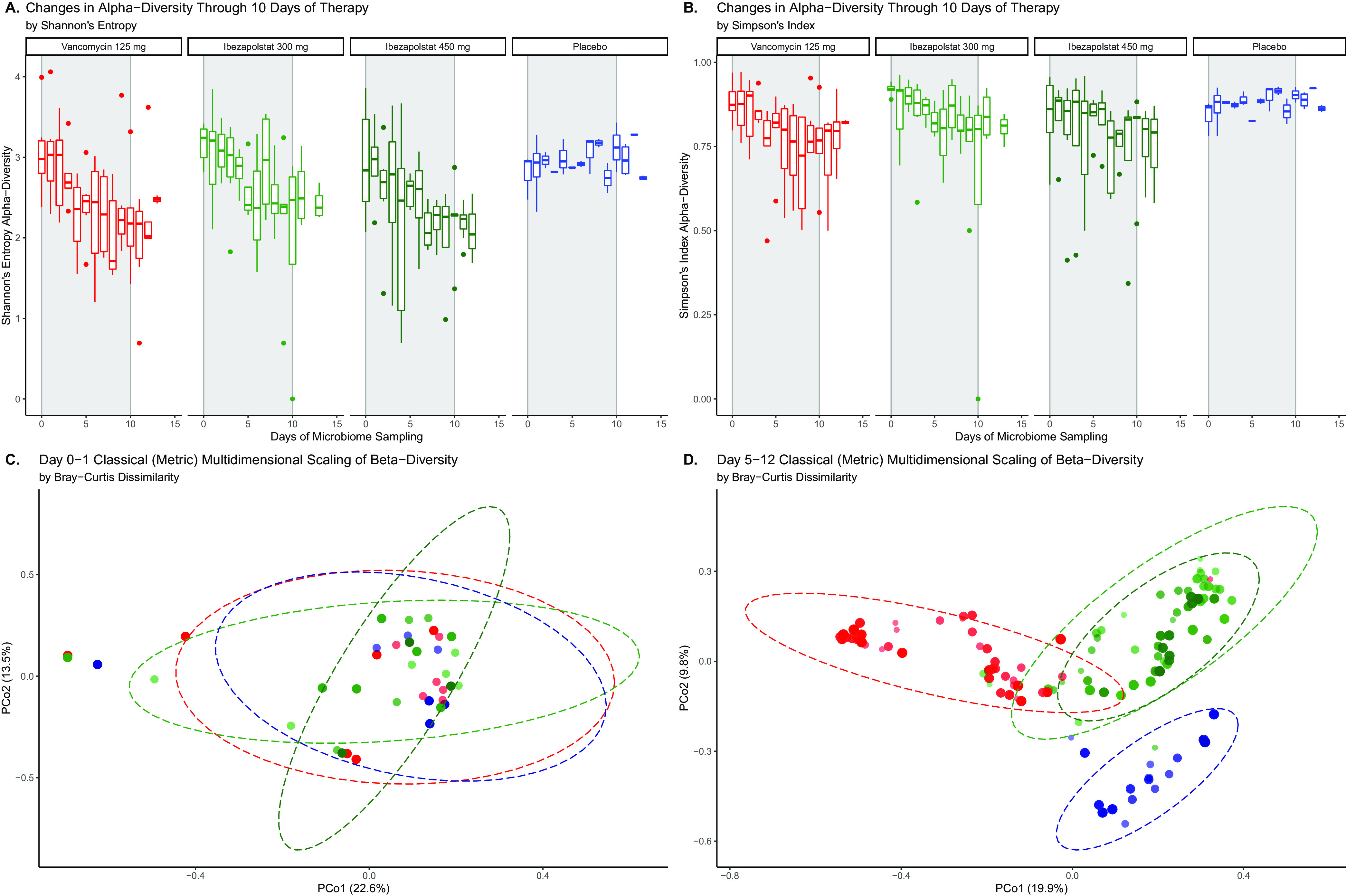
Summary estimates of alpha diversity over time by treatment group, measured by Shannon’s Entropy (A) or Simpson’s Index (B) and beta diversity measured at baseline (C) or after at least 5 days of therapy (D).

**FIG 3:**
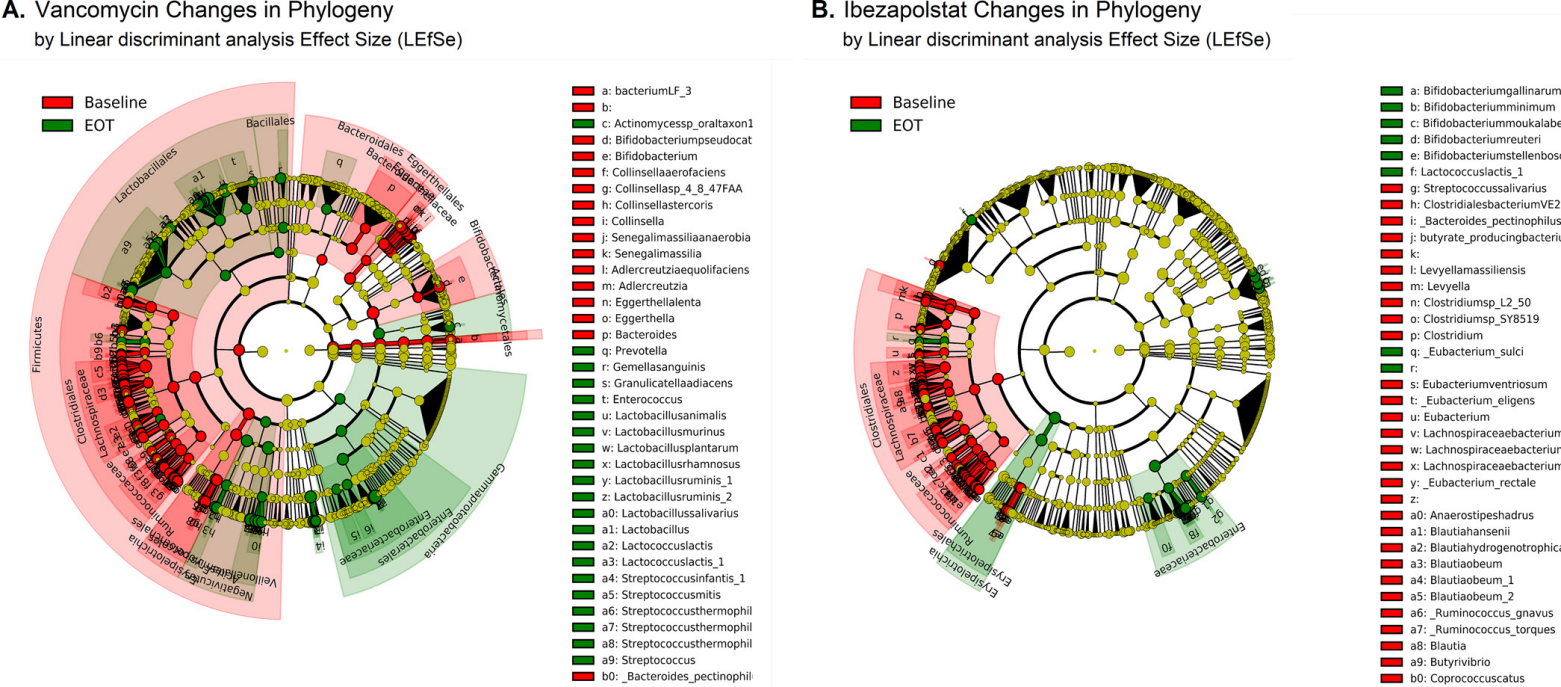
Effects of vancomycin (A) and ibezapolstat (B) on the relative abundance of taxa at baseline versus end of therapy. Significantly higher relative abundance taxa at end of therapy are represented in red, while significantly lower relative abundance taxa at end of therapy are represented in green.

Microbiota were not different at baseline (Day 0 samples) between study groups. The daily changes of individual phyla and Shannon’s index alpha diversity for subjects given ibezapolstat, vancomycin, or the placebo are shown in Fig. 1. Interindividual phylum differences were evident. However, the proportion of Proteobacteria or Fusobacteria increased in subjects given vancomycin, while the proportion of Actinobacteria increased consistently in subjects given ibezapolstat. In general, alpha diversity decreased on therapy for individual subjects who received either ibezapolstat or vancomycin compared to those who received the placebo. A statistical analysis of the changes in alpha diversity over time is shown in Table 1. Using three separate alpha diversity indices (Shannon, Simpson, and Pielous), ibezapolstat 450 mg and vancomycin showed statistically significant changes in alpha diversity over time compared to the placebo. Ibezapolstat 300 mg did not demonstrate statistically significant changes compared to the placebo. Summary measures for alpha diversity changes (Shannon) over time by treatment group is shown in Fig. 2. Beta diversity changes confirmed that microbiota were significantly different between study groups (Fig. 2). A principle coordinate analysis revealed that baseline samples were similar in all study groups, while distinct ellipses representing 95% confidence bounds for each cluster were significantly different for the vancomycin-treated subjects compared to subjects treated with either dosage of ibezapolstat or the placebo. Cladograms at baseline compared to end of therapy, generated by the Linear Effect Size (LEfSe) algorithm, are shown in Fig. 3. Vancomycin had a more wide-ranging effect on the microbiome, including significantly lower proportions of most taxa, except for an increased proportion of Gammaproteobacteria. Ibezapolstat demonstrated a decreased proportion of Clostridiales and increased proportions of Actinobacteria including certain species of Bifidobacteriaceae. Bacterial taxa changes at the phylum, class, order, and family levels are shown in Table 2.

**TABLE 1:** Comparison of (A) daily alpha diversity and (B) bile acid changes during therapy for ibezapolstat versus oral vancomycin^*a*^

Analysis	Ibezapolstat 300 mg (*)	Ibezapolstat 450 mg (*)	Vancomycin 125 mg (*)
A. Alpha diversity analysis			
Shannon	−0.12 ± 0.12 (0.31)	−0.45 ± 0.12 (0.0001)	−0.36 ± 0.11 (0.0014)
Simpson's	−0.013 ± 0.023 (0.59)	−0.072 ± 0.022 (0.0019)	−0.070 ± 0.023 (0.0020)
Pielous	−0.0040 ± 0.024 (0.87)	−0.051 ± 0.024 (0.031)	−0.073 ± 0.024 (0.0016)
B. Bile acid analysis			
1° bile acids, μg/L	−3.7 ± 172 (0.98)	307 ± 161 (0.061)	963 ± 146 (<0.001)
2° bile acids, μg/L	−913 ± 675 (0.18)	−971 ± 629 (0.13)	−1,266 ± 570 (0.030)
1°:2° bile acid ratio	−1.3 ± 4.1 (0.75)	6.2 ± 3.8 (0.11)	19 ± 3.5 (<0.0001)

a Numbers represent average change ± standard deviation over the study time period. A negative (−) number represents decreased (A) diversity or (B) bile acid concentration. 1°: primary; 2°: secondary; *, P value versus placebo, controlling for patient age, weight, and sex.

**TABLE 2:** Proportional changes in taxa in healthy subjects given vancomycin or one of two doses of ibezapolstat^*a*^

	Vancomycin 125 mg		Ibezapolstat 300 mg		Ibezapolstat 450 mg	
Taxa (bold indicates phylum)	Proportional change (mean ± SE)	*P*	Proportional change (mean ± SE)	*P*	Proportional change (mean ± SE)	*P*
**Actinobacteria**	−0.11 ± 0.05	0.032	0.31 ± 0.053	<0.0001	0.31 ± 0.054	<0.0001
c_Actinobacteria	−0.074 ± 0.051	0.14	0.27 ± 0.052	<0.0001	0.29 ± 0.053	<0.0001
c_Actinobacteria o_Bifidobacteriales f_Bifidobacteriaceae	−0.078 ± 0.051	0.1293	0.27 ± 0.053	<0.0001	0.29 ± 0.053	<0.0001
c_Actinobacteria o_Bifidobacteriales	−0.080 ± 0.051	0.1201	0.27 ± 0.053	<0.0001	0.29 ± 0.053	<0.0001
c_Coriobacteriia	−0.038 ± 0.015	0.0145	0.036 ± 0.016	0.0221	0.024 ± 00.16	0.1431
c_Coriobacteriia o_Coriobacteriales	−0.031 ± 0.015	0.0375	0.035 ± 0.016	0.0264	0.026 ± 0.016	0.1013
c_Coriobacteriia o_Coriobacteriales f_Coriobacteriaceae	−0.032 ± 0.015	0.0338	0.034 ± 0.016	0.0298	0.025 ± 0.016	0.1122
**Bacteroidetes**	−0.034 ± 0.024	0.16	−0.0055 ± 0.025	0.83	−0.013 ± 0.025	0.61
**Firmicutes**	−0.14 ± 0.058	0.014	−0.47 ± 0.060	<0.0001	−0.50 ± 0.06	<0.0001
c_Clostridia	−0.50 ± 0.052	<0.0001	−0.49 ± 0.054	<0.0001	−0.52 ± 0.054	<0.0001
c_Clostridia o_Clostridiales	−0.50 ± 0.052	<0.0001	−0.49 ± 0.054	<0.0001	−0.52 ± 0.054	<0.0001
c_Clostridia o_Clostridiales f_Lachnospiraceae	−0.24 ± 0.024	<0.0001	−0.22 ± 0.025	<0.0001	−0.26 ± 0.025	<0.0001
c_Clostridia o_Clostridiales f_Ruminococcaceae	−0.25 ± 0.033	<0.0001	−0.27 ± 0.034	<0.0001	−0.25 ± 0.035	<0.0001
c_Bacilli	0.30 ± 0.043	<0.0001	0.016 ± 0.044	0.72	0.017 ± 0.045	0.39
c_Bacilli o_Lactobacillales	0.30 ± 0.043	<0.0001	0.016 ± 0.044	0.7117	0.017 ± 0.045	0.6972
c_Bacilli o_Lactobacillales f_Lactobacillaceae	0.28 ± 0.041	<0.0001	0.024 ± 0.042	0.5755	0.015 ± 0.043	0.7307
**Fusobacteria**	0.036 ± 0.015	0.0165	0.0011 ± 0.015	0.9414	0.00046 ± 0.015	0.9762
**Proteobacteria**	0.23 ± 0.045	<0.0001	0.12 ± 0.0.5	0.0094	0.09 ± 0.0.5	0.053
c_Gammaproteobacteria	0.21 ± 0.045	<0.0001	0.12 ± 0.046	0.0094	0.092 ± 0.046	0.0478
c_Gammaproteobacteria o_Enterobacterales	0.17 ± 0.042	<0.0001	0.11 ± 0.043	0.0099	0.094 ± 0.044	0.0336
c_Gammaproteobacteria o_Enterobacterales f_Enterobacteriaceae	0.17 ± 0.041	<0.0001	0.11 ± 0.042	0.0082	0.087 ± 0.043	0.043

a c: class; o: order; f: family. Dark gray shading indicates at least a 10% increase in relative proportion compared to baseline, and light gray shading represents a 10% decrease in relative proportion compared to baseline (only variables with a *P* < 0.005 significance colored).

### Bile acids.

**FIG 4:**
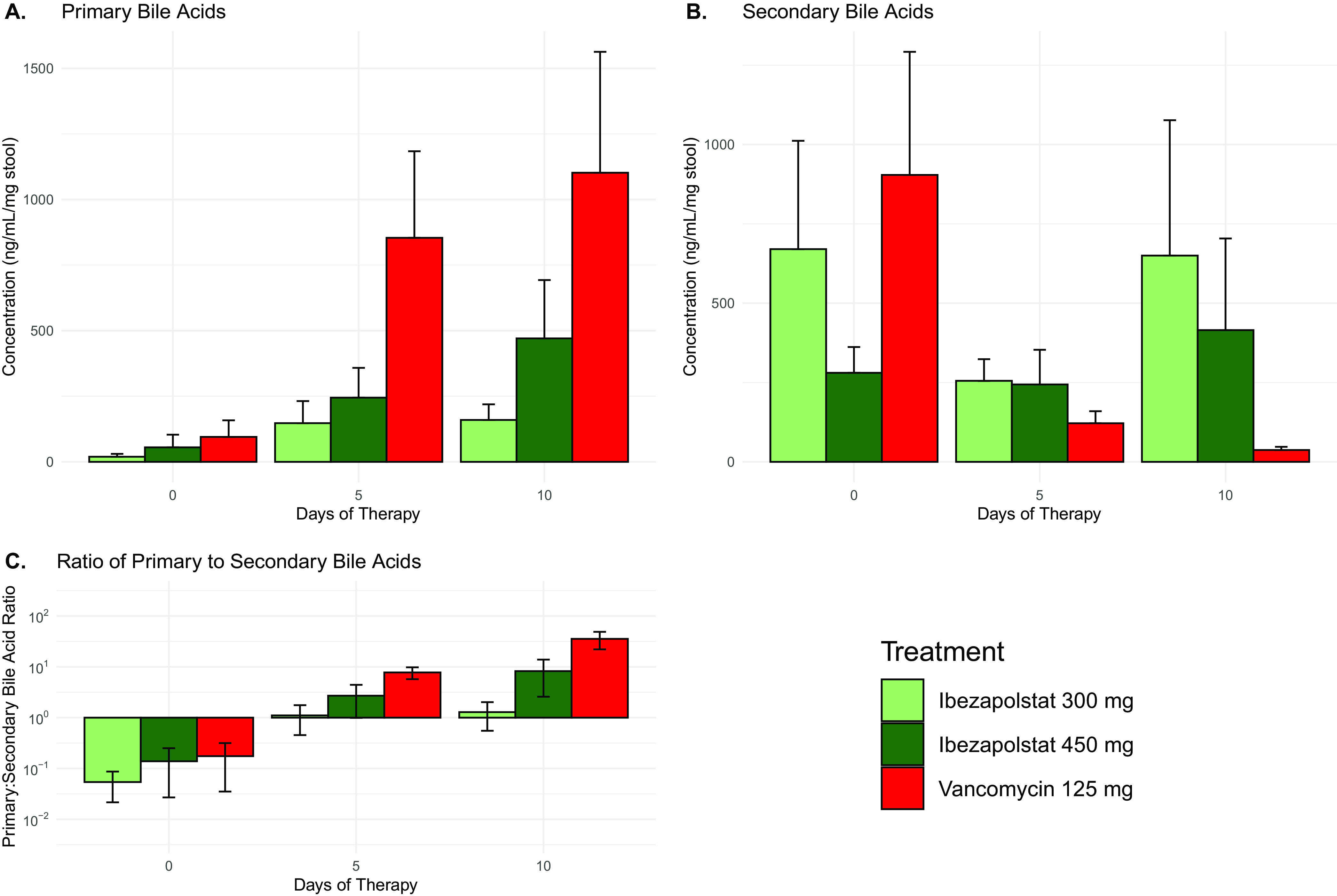
Summary of the changes of primary (A) and secondary (B), as well as the ratio of primary:secondary bile acids (C) over time. Values represent mean ± standard error.

Seventeen baseline samples were available for bile acid analysis along with 17 samples from Day 5 and 14 samples from Day 10. Concentrations of bile acids for each drug and time period are shown in Fig. S1. Baseline samples were similar for all study groups and were comprised primarily (>95%) of secondary bile acids. Primary bile acids increased and secondary bile acids decreased with exposure to all study drugs, but more pronounced findings were observed with vancomycin (Fig. 4). Using a linear regression analysis and after controlling for subject demographics, vancomycin was associated with significant increases in primary bile acids as well as primary:secondary bile acid ratios. Although similar effects were noted with ibezapolstat 450 mg, these results were not statistically significant (Table 1).

### Correlation between microbiota and bile acid changes.

Correlations between family taxa and primary and secondary bile acid concentrations are shown in Table 3. Enterobacteriaceae were most highly correlated with primary bile acid concentrations (*r* = 0.63; *P* < 0.0001) while Ruminococcaceae were negatively correlated with primary bile acid concentrations (*r* = −0.37; *P* = 0.0025). Also, Ruminococcaceae were positively correlated with secondary bile acid concentrations (*r* = 0.44; *P* = 0.0002), and Pseudomonadaceae were positively correlated with secondary bile acid concentrations (*r* = 0.38; *P* = 0.0017).

**TABLE 3:** Correlation of microbiota with bile acids^*a*^

Family	Primary bile acids	*P*	Secondary bile acids	*P*
Bacteroidaceae	−0.20096	0.103	−0.10486	0.3984
Bifidobacteriaceae	−0.07082	0.569	−0.03019	0.8084
Coriobacteriaceae	−0.23574	0.0548	−0.02838	0.8197
**Enterobacteriaceae**	**0.62888**	**<0.0001**	−0.16676	0.1774
Erysipelotrichaceae	−0.12744	0.3041	−0.03216	0.7962
Fusobacteriaceae	−0.0662	0.5946	−0.04921	0.6925
Lachnospiraceae	−0.33184	0.0061	0.01017	0.9349
Lactobacillaceae	0.26868	0.0279	−0.09527	0.4432
Methanobacteriaceae	0.00194	0.9876	−0.01041	0.9333
Pseudomonadaceae	0.27146	0.0263	0.37721	0.006
**Ruminococcaceae**	**−0.36391**	**0.0025**	**0.44424**	**0.0002**

a Boldface entries indicate analyses that were statistically significant.

### BaiCD gene abundance analysis.

**FIG 5:**
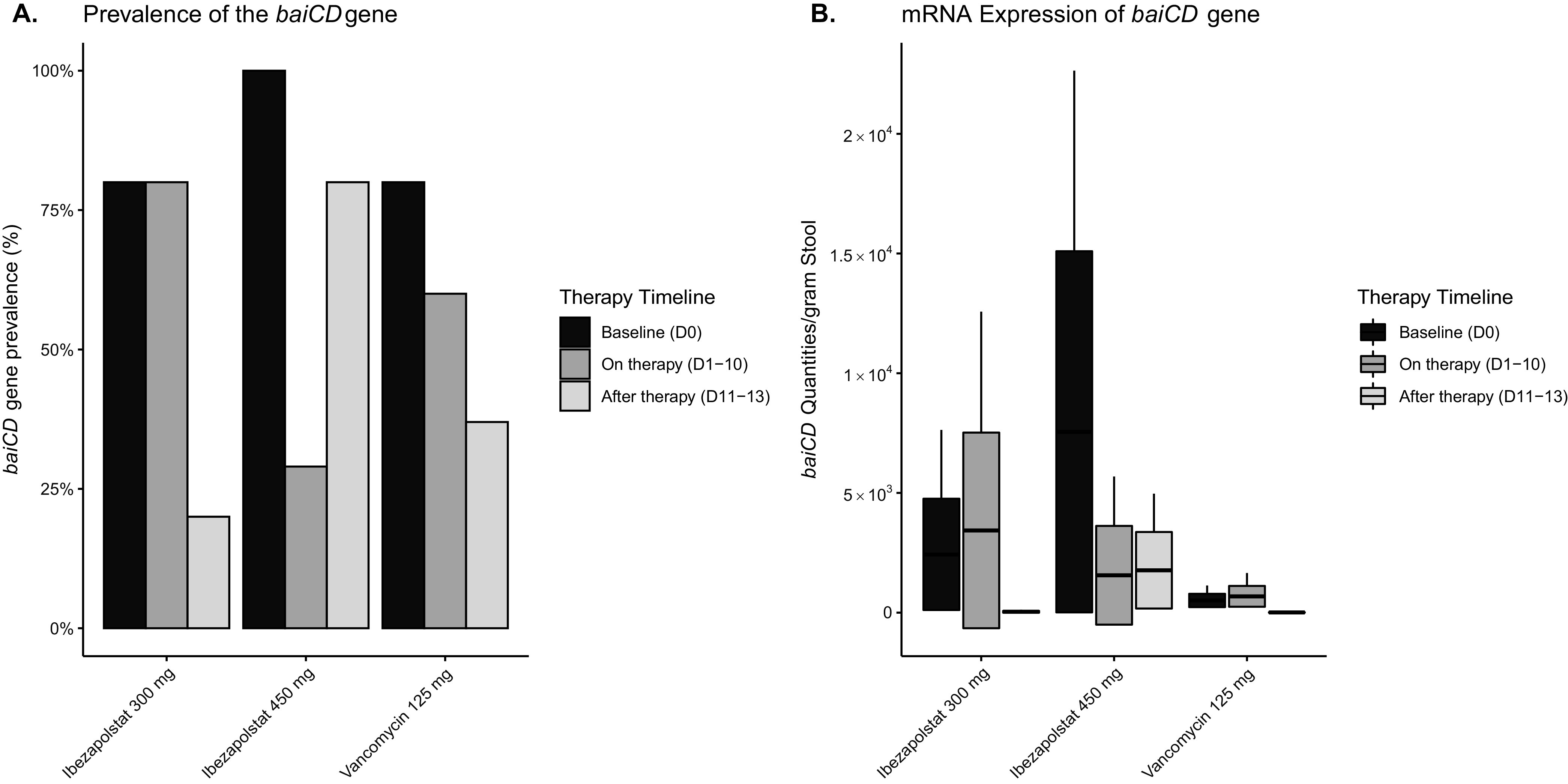
BaiCD gene cluster prevalence and abundance by therapy assignment.

Baseline and follow-up stool samples were available for five patients who received vancomycin and ibezapolstat 450 mg and for four patients that received ibezapolstat 300 mg. The baiCD gene positivity rate was similar between subjects, irrespective of the type of therapy given (80 to 90%). The proportion positive and quantity of baiCD genes decreased during all three types of therapy (Fig. 5).

## DISCUSSION

The pathophysiology of CDI involves disruption of the human gut microbiota, usually with high-risk antibiotics, and can lead to a dysbiosis that enables C. difficile spores to germinate and cause active disease (2). Thus, ideal characteristics for a new drug directed toward CDI include potent activity against C. difficile and minimal further disruption of host microbiota (5). Laboratory and animal models are generally able to identify small molecules with potent *in vitro* activity against C. difficile isolates. However, due to the complex nature of the gut microbiome, identification of a potential ability to reduce the likelihood of CDI recurrence is generally not possible until large Phase III studies are undertaken. This leads to costly and unfortunate mistakes in Phase III clinical studies of novel CDI-directed antibiotics, despite the fact that these antibiotics have different effects on the microbiota than comparator antibiotics do (7, 11). Thus, a method to identify possible anti-recurrence effects earlier in the clinical trial development process is urgently needed. In this study, we used stool samples from the Phase 1 healthy volunteer study to compare ibezapolstat, a DNA polymerase IIIC inhibitor, to vancomycin, a glycopeptide antibiotic and the most commonly used antibiotic to treat CDI, to a placebo. DNA polymerase IIIC inhibitors target low G+C bacteria, namely, Firmicutes, but have no effect on other Gram-positives (Actinobacteria) or Gram-negatives (Bacteroidetes) (13). Alternatively, vancomycin has broad spectrum activity against all of these phyla and would be expected to have a larger effect on the microbiome (14). Using metagenomic sequencing, we confirmed this pharmacology and demonstrated that both antibiotics affected the human microbiome, though they did so in completely distinct manners and produced two distinct microbiome profiles. Using mass spectrometry, we then demonstrated that this change in the microbiome was associated with a reduced effect on the ratio of primary to secondary bile acids in the gut for ibezapolstat compared to that of vancomycin. Family taxa differences observed in subjects given vancomycin or ibezapolstat were highly correlated with concentrations of primary or secondary bile acids. These results are highly suggestive of a possible anti-recurrence effect for ibezapolstat compared to the gold standard, vancomycin. This represents the most thorough evaluation of functional metagenomic changes ever undertaken during Phase I clinical trials for CDI-directed antibiotics. This extends and strengthens the ongoing functional metagenomic work being performed during the Phase II clinical trials of ridinilazole (15, 16). Taken together, these analyses could become a new standard in the drug development process for CDI-directed antibiotics and, in general, could be understood alongside other systemic antibiotics to evaluate their likelihood to increase the risk of CDI with use.

Important advances in the understanding of the pathophysiology of CDI and the mechanisms underlying colonization resistance to C. difficile have transformed our understanding of how certain microbial taxa reduce the likelihood of CDI and recurrent CDI (17). Key metagenomic findings in this study include a consistent decrease in the Clostridia class with both antibiotics, an expansion of the Actinobacteria class in ibezapolstat-treated subjects, and an expansion of the Gammaproteobacteria class, the Enterobacterales order, and the Enterobacteriaceae family in vancomycin-treated subjects. Within the Firmicute phylum, vancomycin was also associated with an increased proportion of Bacilli class taxa. Most metagenomic studies with C. difficile have focused on recurrent CDI and the effect of fecal microbiota transplantation (FMT) (18). An expansion of the Enterobacteriaceae family has been previously identified as a significant risk factor for recurrent CDI (19, 20). FMT studies have also shown that the resolution of CDI recurrence was associated with the restoration of secondary bile acids. An increasing amount of laboratory evidence has helped to further elucidate the importance of bile acids in the pathophysiology of CDI (21, 22). These include findings that the presence of secondary bile acids prevent the germination of C. difficile spores, while primary bile acids increase sporulation. Primary bile acids are metabolized by key taxa in the human gut microbiota by the 7-alpha dehydroxylation pathway, and murine studies have shown that antibiotic treatment leads to a loss of secondary bile acids (23). Prior to conversion to secondary bile acids, primary bile acids are deconjugated by commensal bacteria that possess bile salt hydrolase genes. These genes are present in widely distributed taxa. Thus, it is not surprising that no differences were observed in the proportion of conjugated versus non-conjugated bile acids in our study, despite differences in the microbiome profiles. On the other hand, the 7-alpha dehydroxylation pathways are encoded in the bile acid-inducible (bai) operon. Only a unique set of key species, most commonly Clostridium scindens, Clostridium sordelli, and a small subset of other Firmicutes, are known to possess the full gene for the bai operon (24, 25). Proportions of all of these Clostridiales would be expected to be reduced following vancomycin or ibezapolstat, as demonstrated by changes in baiCD gene abundance during therapy. However, the preservation of secondary bile acids in our study is supported by the Phase II ridinilazole clinical study, in which a similar preservation of secondary bile acids was observed, despite *in vitro* activity of ridinilazole to C. scindens and C. sordelli (15). This suggests that other bacterial taxa also contribute to primary bile acid metabolism (26) or that a group of bacteria that have a subset of the bai pathways could collaboratively synthesize secondary bile acids from conjugated primary bile acids (27). This is a future area of research, but these results suggest that the findings from this study will be applicable to future CDI clinical trials with ibezapolstat. Our plans are to validate and expand these findings in upcoming Phase II studies.

This study has certain limitations. We recruited young, healthy patients into the Phase I clinical trial. The Actinobacteria phylum is more prevalent in younger adults and is replaced by Bacteroidetes with age (28). CDI is more prevalent in older patients, and thus, the baseline microbiota would not be indicative of a healthy microbiome of an elderly patient. However, the Bacteroidetes phylum was present in the majority of our samples and thus was represented as a minority phylum in our study. Whether an expansion of Actinobacteria can be observed in elderly patients with CDI will require further study. If not, an intriguing possibility for a future clinical trial would be to add a probiotic that contains the Actinobacteria phylum to promote Actinobacteria expansion. Likewise, the microbiome of CDI patients may already be characterized by an expansion of Proteobacteria (20). Whether ibezapolstat would be able to reduce this phylum via the expansion of Actinobacteria will require further mechanistic and clinical studies. A common limitation of all human gut microbiome studies is the dependence on daily bowel movements for daily sample collection. As this was not the case for all subjects, samples were not available for each study day for all patients. Lastly, we plan to explore whether these types of analyses could be performed in preclinical, mini-bioreactor models in the future (29).

### Conclusion.

Using data from the Phase 1 healthy volunteer trials and a novel analysis technique, beneficial changes suggestive of a lower risk of CDI recurrence were associated with ibezapolstat compared to vancomycin. This novel functional metagenomics approach may enable the better and earlier prediction of anti-CDI recurrence effects for antibiotics in the clinical development pipeline.

## MATERIALS AND METHODS

### Materials.

Standards for primary bile acids cholate (CA) and chenodeoxycholate (CDCA), conjugated primary bile acids glycocholate (GCA), taurocholate (TCA), glycochenodeoxycholate (GCDCA), and taurochenodeoxycholate (TCDCA), secondary bile acids lithocholate (LCA), deoxycholate (DCA), ursodeoxycholate (UDCA), and hyodeoxycholate (HDCA), and conjugated secondary bile acids glycolithocholate (GLCA), taurolithocholate (TLCA), glycodeoxycholate (GDCA), and taurodeoxycholate (TDCA) were purchased from Sigma.

### Description of clinical trial.

Stool samples were collected daily as part of a recent Phase I healthy volunteer study from the multiday, ascending dose arm that included ibezapolstat (300 or 450 mg, given twice daily) with a vancomycin comparator arm (125 mg four times daily) and a placebo, as described (12). Institutional Review Board approval was obtained (Midlands Institutional Review Board IRB no. 222220170383), and all volunteers signed an informed consent form prior to performing any study procedures. For this analysis, stool samples were collected daily for Days 0 (baseline) to 13, along with a Day 30 follow-up, if available. Stool samples were immediately frozen at –80C prior to shipping to the University of Houston on dry ice for analysis.

### Stool DNA extraction and Shotgun Metagenomic Sequencing.

Stool DNA was extracted using a DNeasy Power Soil Pro Kit (Qiagen, catalog number 1288-100) in a QiaCube automated DNA extraction system, as previously described (12). Shotgun metagenomic sequencing was carried out at the University of Houston Sequencing and Gene Editing Core (Houston, TX, USA) using a Nextera DNA Flex Library Prep Kit for DNA library preparation and an Illumina NextSeq 500 platform for sequencing. CLC Genomic Workbench version 12 (Qiagen) was used for the metagenomic assembly and the creation of the abundance table. Specifically, the tutorial “Taxonomic profiling of whole shotgun metagenomic data” was used to remove host DNA and perform quality control checks. (https://resources.qiagenbioinformatics.com/tutorials/Taxonomic_Profiling.pdf, accessed Mar 28, 2022).

### Extraction of bile acids from stool samples.

Stool samples were aliquoted and weighed (ranging from approximately 10 to 150 mg). Each aliquot was mixed well with 1 mL of 100% methanol containing the internal standards (LCA-d5 and CA-d5, 200 μg/L) by vortexing and ultrasonication. The mixture was placed overnight at 4°C and was centrifuged for 3 min at 10,000 g. The supernatant was transferred into a new tube and diluted 10-fold with pure water. Subsequently, the diluted supernatant was applied to the preconditioned Sep-Pak C18 Classic Cartridge or Waters Corp. Oasis HLB 96-well Plate (Waters, USA). After being washed with 5% methanol, the bile-acid fraction was eluted with 100% methanol. The elution was dried under nitrogen, resuspended in 2 mL of methanol/water (1:1, vol/vol), and stored at −20°C until further analysis was to be completed.

### Bile acid analysis.

Bile acids were quantified using a targeted liquid chromatography mass spectrometry (LC-MS) analysis performed on a QTRAP 5500 mass spectrometer (Sciex, Framingham, MA, USA) adapted from a previously described method (30). Briefly, chromatographic separation between bile acids of similar mass and chemical structures was conducted on a C18 column (Phenomenex, Torrance, CA, USA) via a gradient method using two mobile phases (Solvent A: methanol-water [1:1, vol/vol] with 10 mM ammonium acetate and 0.1% [wt/vol] ammonium hydroxide [pH 9]; Solvent B: methanol with 10 mM ammonium acetate and 0.1% [wt/vol] ammonium hydroxide [pH 9]). Quantification of each type of bile acid was calculated from the standard curves generated using unlabeled and stable isotope-labeled standards of bile acids. Bile acid concentrations were normalized by the corresponding sample weights.

### Bile acid-inducible (bai) gene abundance.

A previously published species-specific quantitative polymerase chain reaction (qPCR), which detected the bai gene abundance present in Clostridium scindens and Clostridium hylemonae (baiCD), was adapted (31). The baiCD analysis was performed using the QuantStudio 5 Real Time PCR System (Applied Biosystems). Also, baiCD gene cluster-specific primers were used, including the forward primer baiCD-F (5′-CAGCCCRCAGATGTTCTTTG -3′) and the reverse primer baiCD-R (5′-GCATGGAATTCHACTGCRTC-3′). The DNA quantity was assessed, and qPCR was performed on each sample in triplicate in a final volume of 20 μL containing 25 ng DNA template, primers at 0.5 μM, and QuantiTech SYBR green Mixes (Qiagen). Threshold cycle values were converted to copies per ng of DNA using a standard curve. Standards were prepared by genomic DNA related to the copy number of Clostridium scindens and a series of serial 10-fold dilutions of the organism DNA. A range of 10-fold serially diluted standard DNA (3 × 10^6^ to 30 copies) was run on each qPCR plate in triplicate. Standard curve *R*^2^ values were calculated for the standards. Copies per gram of stool were calculated, accounting for initial sample DNA concentrations and stool weights.

### Statistical Analysis.

Subject specific and summary changes in bacterial taxa and alpha diversity were generated using the R software package. Linear regression models were built to assess proportional taxa differences at the phylum, class, order, and family levels over time for subjects given vancomycin or ibezapolstat, normalizing to taxa present in at least five percent of the total samples. Linear regression models were also built to assess daily changes in alpha diversity measures (Shannon, Simpson, and Pielous) over time for subjects given vancomycin or ibezapolstat. The LEfSe algorithm was used to visualize and identify significant differences in microbiota composition between baseline samples and Day 10 samples (32). Linear regression models were also built to assess primary and secondary bile acid changes over time as well as the ratio of primary:secondary bile acids over time from subjects given vancomycin or ibezapolstat. All linear regression models used placebo results as baseline values and controlled for subject age, weight, and sex. SAS version 9.4 (SAS Institute, Cary, NC) or R were used for all statistical analyses. The correlation between microbiota and bile acid changes were evaluated at the family taxa for primary and secondary bile acid amounts. To account for multiple analyses per aim and limit the false detection rate, a reduced *P* value of *P* < 0.005 was considered to be indicative of statistical significance (unless otherwise stated) (33).

### Data availability.

All data associated with this study are available in the main text or in the supplemental material. The Illumina paired-end FASTQ files have been deposited in NCBI under BioProject ID PRJNA847068.

## References

[1] Lessa FC, Winston LG, McDonald LC, Emerging Infections Program C. difficile Surveillance Team. 2015. Burden of Clostridium difficile infection in the United States. N Engl J Med 372:2369–2370. doi:10.1056/NEJMc1505190. 26061850 PMC10880113

[2] Britton RA, Young VB. 2014. Role of the intestinal microbiota in resistance to colonization by Clostridium difficile. Gastroenterology 146:1547–1553. doi:10.1053/j.gastro.2014.01.059. 24503131 PMC3995857

[3] Johnson S, Lavergne V, Skinner AM, et al. 2021. Clinical practice guideline by the Infectious Diseases Society of America (IDSA) and Society for Healthcare Epidemiology of America (SHEA): 2021 focused update guidelines on management of Clostridioides difficile infection in adults. Clin Infect Dis 73(5):e1029–e1044. doi:10.1093/cid/ciab549. 34164674

[4] Alam MJ, Walk ST, Endres BT, et al. 2017. Community environmental contamination of toxigenic Clostridium difficile. Open Forum Infect Dis 4(1):ofx018. doi:10.1093/ofid/ofx018. 28480289 PMC5414050

[5] Bassères E, Endres BT, Dotson KM, Alam MJ, Garey KW. 2017. Novel antibiotics in development to treat Clostridium difficile infection. Curr Opin Gastroenterol 33:1–7. doi:10.1097/MOG.0000000000000332. 28134686

[6] Endres BT, Bassères E, Alam MJ, Garey KW. 2017. Cadazolid for the treatment of Clostridium difficile. Expert Opin Invest Drugs 26:509–514. doi:10.1080/13543784.2017.1304538. 28286992

[7] Gerding DN, Cornely OA, Grill S, Kracker H, Marrast AC, Nord CE, Talbot GH, Buitrago M, Gheorghe Diaconescu I, Murta de Oliveira C, Preotescu L, Pullman J, Louie TJ, Wilcox MH. 2019. Cadazolid for the treatment of Clostridium difficile infection: results of two double-blind, placebo-controlled, non-inferiority, randomised phase 3 trials. Lancet Infect Dis 19:265–274. doi:10.1016/S1473-3099(18)30614-5. 30709665

[8] Endres BT, Bassères E, Khaleduzzaman M, Alam MJ, Chesnel L, Garey KW. 2016. Evaluating the effects of surotomycin treatment on Clostridium difficile toxin A and B production, immune response, and morphological changes. Antimicrob Agents Chemother 60:3519–3523. doi:10.1128/AAC.00211-16. 27021314 PMC4879405

[9] Lee CH, Patino H, Stevens C, Rege S, Chesnel L, Louie T, Mullane KM. 2016. Surotomycin versus vancomycin for Clostridium difficile infection: phase 2, randomized, controlled, double-blind, non-inferiority, multicentre trial. J Antimicrob Chemother 71:2964–2971. doi:10.1093/jac/dkw246. 27432604

[10] Boix V, Fedorak RN, Mullane KM, Pesant Y, Stoutenburgh U, Jin M, Adedoyin A, Chesnel L, Guris D, Larson KB, Murata Y. 2017. Primary outcomes from a Phase 3, randomized, double-blind, active-controlled trial of surotomycin in subjects With Clostridium difficile infection. Open Forum Infect Dis 4:ofw275. doi:10.1093/ofid/ofw275. 28480267 PMC5414029

[11] Daley P, Louie T, Lutz JE, Khanna S, Stoutenburgh U, Jin M, Adedoyin A, Chesnel L, Guris D, Larson KB, Murata Y. 2017. Surotomycin versus vancomycin in adults with Clostridium difficile infection: primary clinical outcomes from the second pivotal, randomized, double-blind, Phase 3 trial. J Antimicrob Chemother 72:3462–3470. doi:10.1093/jac/dkx299. 28961905

[12] Garey KW, Begum K, Lancaster C, Gonzales-Luna A, Bui D, Mercier J, Seng Yue C, Ducharme MP, Hu M, Vince B, Silverman MH, Alam MJ, Kankam M. 2020. A randomized, double-blind, placebo-controlled, single and multiple ascending dose Phase 1 study to determine the safety, pharmacokinetics and food and faecal microbiome effects of ibezapolstat administered orally to healthy subjects. J Antimicrob Chemother 75:3635–3643. doi:10.1093/jac/dkaa364. 32892222 PMC7662179

[13] Xu W-C, Silverman MH, Yu XY, Wright G, Brown N. 2019. Discovery and development of DNA polymerase IIIC inhibitors to treat Gram-positive infections. Bioorg Med Chem 27:3209–3217. doi:10.1016/j.bmc.2019.06.017. 31221610

[14] Schubert AM, Sinani H, Schloss PD. 2015. Antibiotic-induced alterations of the murine gut microbiota and subsequent effects on colonization resistance against Clostridium difficile. mBio 6:e00974. doi:10.1128/mBio.00974-15. 26173701 PMC4502226

[15] Qian X, Yanagi K, Kane AV, Alden N, Lei M, Snydman DR, Vickers RJ, Lee K, Thorpe CM. 2020. Ridinilazole, a narrow spectrum antibiotic for treatment of Clostridioides difficile infection, enhances preservation of microbiota-dependent bile acids. Am J Physiol Gastrointest Liver Physiol 319:G227–G237. doi:10.1152/ajpgi.00046.2020. 32597706 PMC7500266

[16] Thorpe CM, Kane AV, Chang J, Tai A, Vickers RJ, Snydman DR. 2018. Enhanced preservation of the human intestinal microbiota by ridinilazole, a novel Clostridium difficile-targeting antibacterial, compared to vancomycin. PLoS One 13:e0199810. doi:10.1371/journal.pone.0199810. 30071046 PMC6071993

[17] Taur Y, Pamer EG. 2014. Harnessing microbiota to kill a pathogen: fixing the microbiota to treat Clostridium difficile infections. Nat Med 20:246–247. doi:10.1038/nm.3492. 24603796 PMC4542075

[18] Mullish BH, Allegretti JR. 2021. The contribution of bile acid metabolism to the pathogenesis of Clostridioides difficile infection. Therap Adv Gastroenterol 14:175628482110177. doi:10.1177/17562848211017725. PMC8165815 34104212

[19] Martinez-Gili L, McDonald JAK, Liu Z, Kao D, Allegretti JR, Monaghan TM, Barker GF, Miguéns Blanco J, Williams HRT, Holmes E, Thursz MR, Marchesi JR, Mullish BH. 2020. Understanding the mechanisms of efficacy of fecal microbiota transplant in treating recurrent Clostridioides difficile infection and beyond: the contribution of gut microbial-derived metabolites. Gut Microbes 12:1810531. doi:10.1080/19490976.2020.1810531. 32893721 PMC7524310

[20] Allegretti JR, Kearney S, Li N, Bogart E, Bullock K, Gerber GK, Bry L, Clish CB, Alm E, Korzenik JR. 2016. Recurrent Clostridium difficile infection associates with distinct bile acid and microbiome profiles. Aliment Pharmacol Ther 43:1142–1153. doi:10.1111/apt.13616. 27086647 PMC5214573

[21] Theriot CM, Bowman AA, Young VB. 2016. Antibiotic-induced alterations of the gut microbiota alter secondary bile acid production and allow for Clostridium difficile spore germination and outgrowth in the large intestine. mSphere 1:e00045. doi:10.1128/mSphere.00045-15. PMC4863611 27239562

[22] Francis MB, Allen CA, Shrestha R, Sorg JA. 2013. Bile acid recognition by the Clostridium difficile germinant receptor, CspC, is important for establishing infection. PLoS Pathog 9:e1003356. doi:10.1371/journal.ppat.1003356. 23675301 PMC3649964

[23] Theriot CM, Koenigsknecht MJ, Carlson PE, Hatton GE, Nelson AM, Li B, Huffnagle GB, Z Li J, Young VB. Jr., 2014. Antibiotic-induced shifts in the mouse gut microbiome and metabolome increase susceptibility to Clostridium difficile infection. Nat Commun 5:3114. doi:10.1038/ncomms4114. 24445449 PMC3950275

[24] Buffie CG, Bucci V, Stein RR, McKenney PT, Ling L, Gobourne A, No D, Liu H, Kinnebrew M, Viale A, Littmann E, van den Brink MRM, Jenq RR, Taur Y, Sander C, Cross JR, Toussaint NC, Xavier JB, Pamer EG. 2015. Precision microbiome reconstitution restores bile acid mediated resistance to Clostridium difficile. Nature 517:205–208. doi:10.1038/nature13828. 25337874 PMC4354891

[25] Ridlon JM, Devendran S, Alves JM, Doden H, Wolf PG, Pereira GV, Ly L, Volland A, Takei H, Nittono H, Murai T, Kurosawa T, Chlipala GE, Green SJ, Hernandez AG, Fields CJ, Wright CL, Kakiyama G, Cann I, Kashyap P, McCracken V, Gaskins HR. 2020. The 'in vivo lifestyle' of bile acid 7alpha-dehydroxylating bacteria: comparative genomics, metatranscriptomic, and bile acid metabolomics analysis of a defined microbial community in gnotobiotic mice. Gut Microbes 11:381–404. doi:10.1080/19490976.2019.1618173. 31177942 PMC7524365

[26] Vital M, Rud T, Rath S, Pieper DH, Schlüter D. 2019. Diversity of bacteria exhibiting bile acid-inducible 7alpha-dehydroxylation genes in the human gut. Comput Struct Biotechnol J 17:1016–1019. doi:10.1016/j.csbj.2019.07.012. 31428294 PMC6692061

[27] Heinken A, Ravcheev DA, Baldini F, Heirendt L, Fleming RMT, Thiele I. 2019. Systematic assessment of secondary bile acid metabolism in gut microbes reveals distinct metabolic capabilities in inflammatory bowel disease. Microbiome 7:75. doi:10.1186/s40168-019-0689-3. 31092280 PMC6521386

[28] Stewart CJ, Ajami NJ, O'Brien JL, Hutchinson DS, Smith DP, Wong MC, Ross MC, Lloyd RE, Doddapaneni H, Metcalf GA, Muzny D, Gibbs RA, Vatanen T, Huttenhower C, Xavier RJ, Rewers M, Hagopian W, Toppari J, Ziegler A-G, She J-X, Akolkar B, Lernmark A, Hyoty H, Vehik K, Krischer JP, Petrosino JF. 2018. Temporal development of the gut microbiome in early childhood from the TEDDY study. Nature 562:583–588. doi:10.1038/s41586-018-0617-x. 30356187 PMC6415775

[29] Gonzales-Luna AJ, Spinler JK, Oezguen N, Khan MAW, Danhof HA, Endres BT, Alam MJ, Begum K, Lancaster C, Costa GP, Savidge TC, Hurdle JG, Britton R, Garey KW. 2021. Systems biology evaluation of refractory Clostridioides difficile infection including multiple failures of fecal microbiota transplantation. Anaerobe 70:102387. doi:10.1016/j.anaerobe.2021.102387. 34044101 PMC8384661

[30] Scherer M, Gnewuch C, Schmitz G, Liebisch G. 2009. Rapid quantification of bile acids and their conjugates in serum by liquid chromatography-tandem mass spectrometry. J Chromatogr B Analyt Technol Biomed Life Sci 877:3920–3925. doi:10.1016/j.jchromb.2009.09.038. 19819765

[31] Solbach P, Chhatwal P, Woltemate S, Tacconelli E, Buhl M, Gerhard M, Thoeringer CK, Vehreschild MJGT, Jazmati N, Rupp J, Manns MP, Bachmann O, Suerbaum S. 2018. BaiCD gene cluster abundance is negatively correlated with Clostridium difficile infection. PLoS One 13:e0196977. doi:10.1371/journal.pone.0196977. 29738579 PMC5940204

[32] Segata N, Izard J, Waldron L, Gevers D, Miropolsky L, Garrett WS, Huttenhower C. 2011. Metagenomic biomarker discovery and explanation. Genome Biol 12:R60. doi:10.1186/gb-2011-12-6-r60. 21702898 PMC3218848

[33] Korthauer K, Kimes PK, Duvallet C, Reyes A, Subramanian A, Teng M, Shukla C, Alm EJ, Hicks SC. 2019. A practical guide to methods controlling false discoveries in computational biology. Genome Biol 20:118. doi:10.1186/s13059-019-1716-1. 31164141 PMC6547503

