# Supplementary material for: Functional and Metagenomic Evaluation of Ibezapolstat for Early Evaluation of Anti-Recurrence Effects in Clostridioides difficile Infection
Source: Antimicrob Agents Chemother. 2022 Jul 6;66(8):e02244-21. doi: 10.1128/aac.02244-21 (PMC9380534; doi:10.1128/aac.02244-21)

Supplemental Figure 1. Bile acid concentrations from IBZ- and VAN-treated subjects at baseline, mid-point of therapy, and end-of-therapy

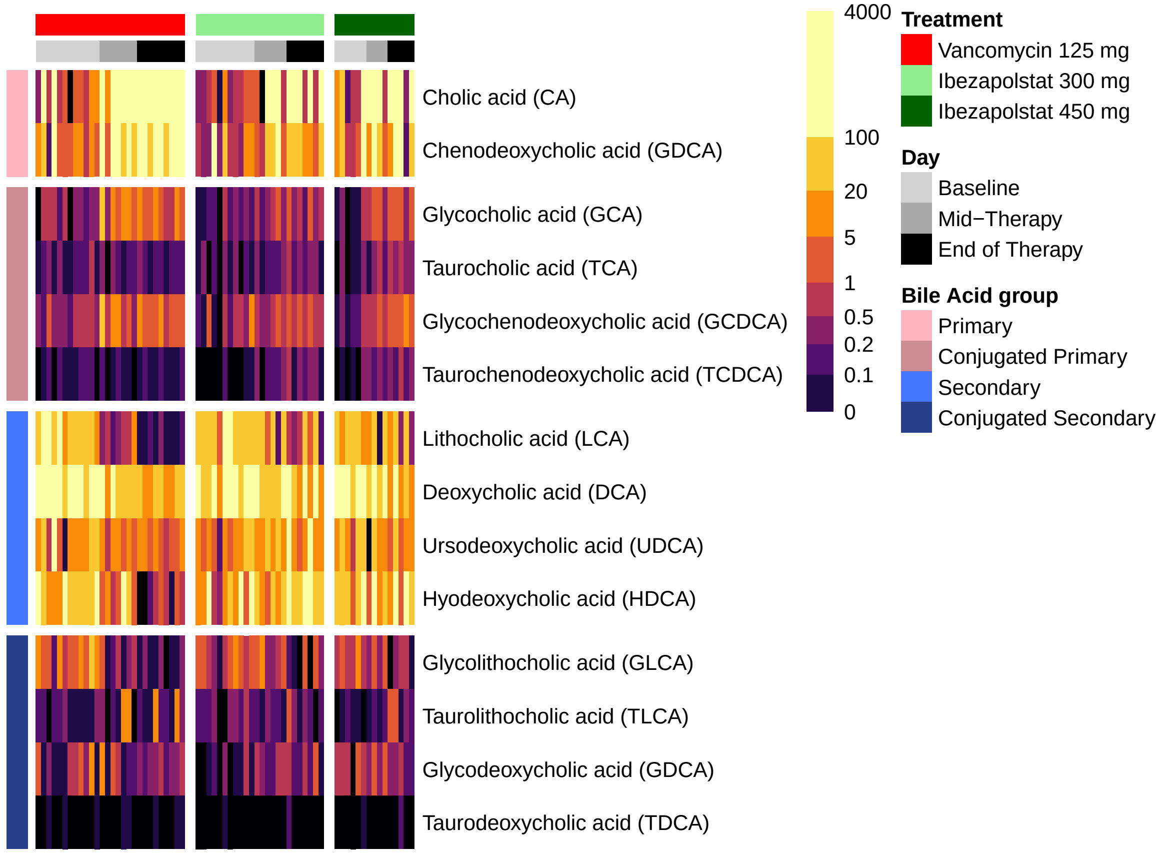

Supplement: Supplemental file 1 — Fig. S1. Download aac.02244-21-s0001.pdf, PDF file, 0.04 MB [file aac.02244-21-s0001.pdf]
